# Revisiting decade-old questions in proanthocyanidin biosynthesis: current understanding and new challenges

**DOI:** 10.3389/fpls.2024.1373975

**Published:** 2024-03-26

**Authors:** Nan Lu

**Affiliations:** BioDiscovery Institute and Department of Biological Sciences, University of North Texas, Denton, TX, United States

**Keywords:** proanthocyanidins, condensed tannins, anthocyanin, anthocyanidin reductase, leucocyanidin reductase

## Abstract

Proanthocyanidins (PAs), one of the most abundant natural polymers found in plants, are gaining increasing attention because of their beneficial effects for agriculture and human health. The study of PA biosynthesis has been active for decades, and progress has been drastically accelerated since the discovery of key enzymes such as Anthocyanidin Reductase (ANR), Leucoanthocyanidin Reductase (LAR), and key transcription factors such as Transparent Testa 2 (TT2) and Transparent Testa 8 (TT8) in the early 2000s. Scientists raised some compelling questions regarding PA biosynthesis about two decades ago in the hope that addressing these questions would lead to an enhanced understanding of PA biosynthesis in plants. These questions focus on the nature of starter and extension units for PA biosynthesis, the stereochemistry of PA monomers and intermediates, and how and where the polymerization or condensation steps work subcellularly. Here, I revisit these long-standing questions and provide an update on progress made toward answering them. Because of advanced technologies in genomics, bioinformatics and metabolomics, we now have a much-improved understanding of functionalities of key enzymes and identities of key intermediates in the PA biosynthesis and polymerization pathway. Still, several questions, particularly the ones related to intracellular PA transportation and deposition, as well as enzyme subcellular localization, largely remain to be explored. Our increasing understanding of PA biosynthesis in various plant species has led to a new set of compelling open questions, suggesting future research directions to gain a more comprehensive understanding of PA biosynthesis.

## 1 Introduction

**Figure 1:**
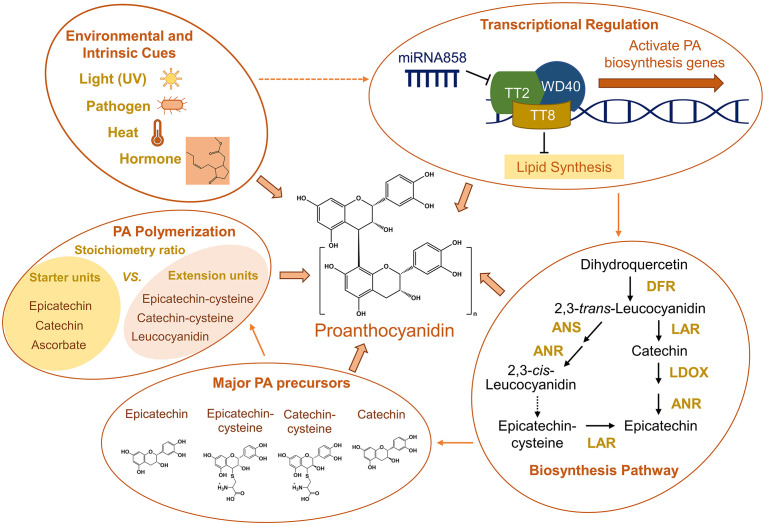
Summary of environmental and intrinsic cues for PA biosynthesis, transcriptional regulation of PA biosynthesis, simplified conventional PA biosynthesis pathway based on studies in Medicago, major PA precursors, and factors affecting PA polymerization. Arrows between groups indicate cause-and-effect relationships. Dashed arrows between groups and in the PA biosynthesis pathway indicate processes that remain unclear. The hormone structure in the “Environmental and Intrinsic Cues” group represents methyl jasmonate. ANR, anthocyanidin reductase; ANS, anthocyanidin synthase; DFR, dihydroflavonol-4-reductase; LAR, leucoanthocyanidin reductase; LDOX, leucoanthocyanidin dioxygenase.

Proanthocyanidins (PAs), or condensed tannins (CTs), are oligomers of flavan 3-ols naturally produced in plants. Like other polyphenols, PAs have shown promise of health benefits because of their antioxidant activity. PAs, in particular, are believed to have antidiabetic and anticancer functions and have beneficial effects in preventing cardiovascular disease and reducing inflammation (Cos et al., 2004). Many plant-based foods and drinks, such as rice, sorghum, soybean, persimmon, grapes, tea and fruit juice, are rich in PAs, making plants an important dietary source of PAs for humans (Bhagwat and Haytowitz, 2015). PAs, while mostly accumulated in seed coats, are found in almost all tissues, including flowers, leaves, stems and roots, where they play a pivotal role in protecting plants from UV damage, abiotic stresses, as well as pest and fungal attack (Dixon and Sarnala, 2020; **Figure 1**). In agriculture, animal feeds are often supplemented with PAs to reduce ruminal bloat, a lethal and costly disease, and to decrease methane emissions from ruminants, a contributing factor to global warming (Waghorn, 2008; Jonker and Yu, 2016).

The beneficial effects of PAs in human and animal health, as well as their role in plant stress responses, have made decoding the PA biosynthesis pathways a research area of interest. Initial progress was made mainly by characterizing mutants with disrupted PA accumulation in PA-rich plants such as barley (*Hordeum vulgare*) and *Arabidopsis thaliana*. The first breakthrough discovery came when Anthocyanidin Reductase (ANR), the enzyme that converts anthocyanidins to flavan 3-ols, was identified and characterized by Xie et al. (2003) in Arabidopsis, which specified the PA-specific branch in the general flavonoid biosynthesis pathway. Shortly after, two review papers (Dixon et al., 2005; Xie and Dixon, 2005) raised a series of open questions that were critical for advancing our understanding of PA biosynthesis. These questions range from functions of some key enzymes in the pathway to the stereochemistry of PA precursors, as well as how and where PAs are transported.

Now, almost two decades since these PA review papers were published, I revisit the original questions, summarize major advances made since then, and come up with a new set of compelling questions, hoping to shed light on future studies toward a better understanding of the regulation and mechanism of PA biosynthesis in plants.

## 2 Milestones in understanding PA biosynthesis

### 2.1 The extended functions of key enzymes in PA biosynthesis in diverse plant species

In PA-rich plant species, ANR is fundamental in making the building blocks (i.e., flavan 3-ols) for PA biosynthesis (**Figure 1**). Loss of activity of ANR results in loss of PAs and increase of anthocyanins in Arabidopsis, *Medicago truncatula*, cotton (*Gossypium hirsutum*) and soybean (*Glycine max*) (Xie et al., 2003; Kovinich et al., 2012; Zhu et al., 2015). Further studies of PA biosynthesis demonstrated that the starter and extension units for PA polymerization are actually generated by two parallel pathways, both of which require the participation of ANR (Jun et al., 2021). In Medicago, the branch leading to epicatechin starter units also involves Leucoanthocyanidin Reductase (LAR) and Leucoanthocyanidin Dioxygenase (LDOX), while the other branch leading to PA extension units requires Anthocyanidin Synthase (ANS) (Jun et al., 2018; **Figure 1**). Intriguingly, many plant species, such as Arabidopsis, seem to only have ANS or LDOX (i.e. LAR is not present), suggesting the complexity and diversity of the PA biosynthesis pathway in various plant species.

In wheat (*Triticum aestivum*) and maize (*Zea mays*), two species that do not accumulate PAs, ANRs preferably produce (+)-epicatechin rather than the (-)-epicatechin stereoisomer commonly found in PA-rich plants, suggesting that ANRs in wheat and maize may have evolved distinct functions and may contribute to the lack of PA oligomers in these two species (Jun et al., 2021; Lu et al., 2023).

As mentioned before, one of the key enzymes at the branch point of PA biosynthesis is LAR, which was initially known for catalyzing the synthesis of catechin, a starter unit for PA polymerization (Bogs et al., 2005). A decade later, a study of PA biosynthesis in Medicago suggested a role of LAR in balancing the ratio of PA starter and extension units (Liu et al., 2016; **Figure 1**). Loss of LAR activity in Medicago seeds led to significantly increased levels of insoluble (highly polymerized) PAs and reduced levels of soluble PAs, indicating that the LAR-dependent ratio of starter and extension units is a crucial factor for determining the degree of PA polymerization. However, as mentioned above, there is no evidence so far suggesting the existence of any LAR-like enzymes in some PA-rich plant species like Arabidopsis, raising the question of how PA chain length is controlled in these species.

### 2.2 Identification of key intermediates in PA biosynthesis

Identification and characterization of intermediates in the biosynthesis of PA monomers has been a focal point of research interest, as the reaction intermediates are key to understanding the mechanistic details of PA biosynthesis. While much effort has been made to search for intermediates in PA biosynthesis, only a few compounds, such as 4β-(S-cysteinyl)-epicatechin and 2,3-*cis*-leucocyanidin, have been identified so far as possible intermediates in PA monomer biosynthesis and PA polymerization (Liu et al., 2016; Wang et al., 2020; Jun et al., 2021; Yu et al., 2022; **Figure 1**). Identifying intermediates in PA biosynthesis reactions can be challenging, because of the instability of flavonoid carbocations. Thus, developing new approaches that can effectively “capture” these compounds will be key to elucidating the mechanisms of PA biosynthesis in plants.

### 2.3 The role of TT19 (AtGSTF12) beyond anthocyanin deposition

While TT19 has long been considered involved in both anthocyanin and PA biosynthesis, the exact function of TT19 beyond the role in anthocyanin deposition has been a mystery ever since its homologous genes were first discovered in maize (McLaughlin and Walbot, 1987) and petunia (Alfenito et al., 1998). It was proposed the oxidized anthocyanins were the reason for the bronze phenotype in maize seeds (Marrs et al., 1995). In Arabidopsis *tt19* mutant, besides the loss of anthocyanins in the stem tissue, its seeds show small vacuoles, and the mechanism causing this phenotype is not clear (Kitamura et al., 2010). In the last few years, progresses have been achieved in understanding the role of TT19 in PA biosynthesis. Loss of TT19 activity in Arabidopsis seeds leads to significant reduction of PA starter units (i.e., epicatechin), but not PA extension units (i.e., epicatechin-cysteine), which consequently results in disrupted ratio of soluble and insoluble PAs (Lu et al., 2022). Another study suggests that TT19-like enzymes possess catalytic functions *in vitro* (Eichenberger et al., 2023). It will be interesting to further investigate how this new role helps explain the TT19 function in PA biosynthesis, and how this mechanism is correlated with the altered vacuole phenotype in the *tt19* mutant.

### 2.4 Engineering PAs in crops

The beneficial effects of PAs in health and agriculture have attracted increasing attention of plant scientists to engineering PAs in crops. As more regulatory factors and key enzymes of the PA biosynthesis pathway are identified and characterized, a number of strategies have been developed to engineer economic crops for enhanced PA production. The most straightforward approach to increase PA production is manipulating master transcription factors regulating the expression of PA biosynthesis genes. Similar to the regulatory machinery controlling anthocyanin biosynthesis, a conserved complex involving multiple classes (MYB, bHLH, WD40 etc.) of transcription factors activates PA biosynthesis genes and initiates PA biosynthesis by binding to promoters of genes in both early and late stages of the pathway (**Figure 1**). Besides, WRKY family of TFs have been shown to involve in the transport of PAs in Arabidopsis and grape (Gonzalez et al., 2016; Amato et al., 2017). Other transcriptional activators and repressors can influence the stability of the complex or its ability to bind on promoters, determining when and where PAs are synthesized in plants (Jun et al., 2015).

Ectopic expression of TaMYB14-1, a TT2-type transcription factor from *Trifolium arvense* (Hancock et al., 2012), in white clover (*Trifolium repens*) significantly improved the level of soluble PAs to over 2% of dry matter, and the PAs in white clover leaves were able to bind to forage proteins and reduce ammonia and methane emissions (Roldan et al., 2022). Cotton plants over-expressing *GhTT2L-3A* produced brown-colored fibers accumulating substantial amounts of PAs, and the fiber quality was also improved in transgenic plants compared to wild-type control plants (Yan et al., 2018). Given that the increased accumulation of PAs in some tissues may cause negative effects on plant growth and development, new approaches using tissue-specific promotors have been proposed for precisely controlled accumulation of PAs (Cui et al., 2022). These achievements in engineering PAs will likely inspire future studies to develop novel strategies for improving PA production in other economically useful crops.

## 3 Rising questions to be addressed and future perspectives

### 3.1 What is the next model species for studying PA biosynthesis?

Arabidopsis and Medicago have long been used as model species to study anthocyanin and PA biosynthesis. This was driven by the early release of their genomes, the generation of tens-of-thousands of mutants, and the easy-to-observe PA phenotype since the wild-type plants naturally accumulate colored PAs in seeds. However, it has come to our attention that there is no one-model-fits-all for PA biosynthesis. Getting knowledge from more species is key to better understanding the “core” enzymes and the “expendable” enzymes in the pathway. With more genomes becoming available on a monthly basis and the transformation efficiency dramatically increasing with new technology emerging, the pool for finding the next model plant species is getting larger. As previously mentioned by Dixon and Sarnala (2020), poplar (*Populus tremula*) is one good candidate model species, because, among other reasons listed, PAs naturally accumulate in various tissues rather than being limited in seeds. Another candidate would be cotton, which is an economically important crop worldwide. Similar to poplar, cotton accumulates PAs in various tissues, including seeds, fibers, leaves, and stems (Lu et al., 2017). In addition, a highly efficient Virus-Induced-Gene-Silencing (VIGS) system makes it easy to test the function of PA-related genes in a timely manner (Zhu et al., 2015). These technologies could also apply to major fruit crops with PA and/or anthocyanin presence such as strawberry and grape (Xie et al., 2020; Yang et al., 2022).

In contrast to the extensively studied PA biosynthesis in dicots, details about the PA biosynthesis pathway and its regulatory mechanism in monocots remain largely unknown with only a few candidate genes identified so far. In rice (*Oryza sativa*), *Rc* and *Rd* were identified as genes encoding a bHLH-type transcription factor and a dihydroflavonol-4-reductase (DFR), respectively (Furukawa et al., 2007). The *tannin1* locus in sorghum encodes a WD40 protein that belongs to the transcription factor ternary complex necessary for activating PA biosynthesis in sorghum (Wu et al., 2012). In barley (*Hordeum vulgare*), the *ant13*, *ant17* and *ant18* loci encode WD40, flavanone 3-hydroxylase (F3H), and DFR, respectively (Olsen et al., 1993; Himi and Taketa, 2015; Shoeva et al., 2023). Notably, those genes identified from mutants with significant PA deficiency phenotypes are either major transcriptional regulators or enzymes at relatively early steps of the PA biosynthetic pathway. Despite these discoveries, the lack of an accessible and saturated mutant pool is still the bottleneck for studying PA biosynthesis in monocots. A recent release of an ethyl methane sulfonate (EMS)-induced sorghum mutant library (Jiao et al., 2023) offers another promising opportunity for studying PA biosynthesis and regulation in monocots.

### 3.2 Will PAs exist in other non-traditional forms?

Because of the reactive nature of the PA precursors, it is not surprising that PAs exist in various forms of oligomers and conjugates. In maize, some purple-colored seeds accumulate anthocyanin-catechin conjugates (Lu et al., 2023). In Arabidopsis *ans* mutant seeds, *trans*-leucocyanidin, as extension units, attacks ascorbate to form catechin-ascorbate oligomers (Yu et al., 2022). Recently, a new form of PA-like oligomers involving flavan 3-ols, named papanridin, was discovered by Zhu et al. (2023). It will be interesting to find out whether additional non-traditional PA-like oligomers or polymers exist in different plant species, and more importantly, to demonstrate the role of these compounds in plants and their beneficial bioactivities.

### 3.3 What determines the level of PA polymerization?

PA polymerization in Arabidopsis and Medicago is currently considered as a spontaneous process that does not require enzyme catalyzation. However, the level of PA polymerization can be affected by many factors, such as the stoichiometry of starter and extension units and the stereochemistry of PA monomers (Liu et al., 2016; Lu et al., 2023). It will be a necessary next step to learn how PA polymerization is determined and how to regulate the level of PA polymerization for different application purposes. Since the PA starter units and extension units are generated in two separate pathways, it would be interesting to know whether it is possible to manipulate enzymes in one branch of the pathway but not the other branch, and whether it is possible to control how and when extension units “find” starter units.

### 3.4 What are other factors affecting PA biosynthesis?

Some well-studied transcription factors (e.g., TT2, TTG1, TT8) can activate or repress the expression of PA-related genes and subsequently affect PA and/or anthocyanin biosynthesis (Lu et al., 2021). Are there other factors that can turn on or off these transcription factors? Recently, several microRNAs that target PA-related transcription factors or enzymes have been identified in grape berry (Vale et al., 2021), apple (*Malus domestica*) (Zhang et al., 2022), persimmon (Yang et al., 2020), kiwifruit (*Actinidia deliciosa*) (Wang et al., 2023) and cotton (Mei et al., 2023). Notably, the miR858 family members target TT2-type MYBs, a key activating regulator of PA biosynthesis functionally conserved in many plant species (**Figure 1**). The involvement of these small RNAs in PA biosynthesis opens a new avenue for PA engineering in crops.

Plant hormones participate in almost all aspects of plant growth and development, but whether or not they play a role in PA biosynthesis remain largely unknown. Recent studies indicated that plant hormones could affect PA biosynthesis. Applying methyl jasmonate induced PA accumulations in apple calli by influencing the interactions between Jasmonate ZIM-domain (MdJAZ) proteins and the MYB-bHLH-WD40 transcription factor complex that regulates PA biosynthesis (An et al., 2015; **Figure 1**). It will be interesting to find out whether and how other families of plant hormones might be involved in regulating PA accumulation in plants.

### 3.5 How is PA biosynthesis related to lipid metabolism?

TT8 has been known for its role in the transcriptional regulation of PA biosynthesis, as disruption of TT8 results in loss of PA in Arabidopsis seed coats (Nesi et al., 2000). A study of lipids in the Arabidopsis *tt8* mutant showed that the accumulation of fatty acids was significantly enhanced in *tt8* seeds, and further transcript analysis showed that TT8 might function as a repressor to down-regulate genes required for lipid biosynthesis (Chen et al., 2014; **Figure 1**). Later on, similar enhanced lipid accumulation phenotypes were observed in seeds of *Brassica napus* and tobacco (*Nicotiana tabacum*) when TT8-like genes were disrupted (Zhai et al., 2020; Tian et al., 2021). These findings suggest crosstalk between flavonoid biosynthesis and central metabolism pathways. Future studies of the crosstalk between PA and lipid biosynthesis may focus on deciphering the regulatory mechanism for maintaining the homeostasis of PAs and lipids, exploring its biological significance in plant growth and development, and developing new strategies for PA and lipid engineering in crops.

## 4 Concluding remarks

Unlike many other metabolite biosynthesis pathways that are conserved among plants, the pathways of PA biosynthesis are complex and divergent in different plant species. Substantial progresses have been made over the past two decades in advancing our understanding of the PA biosynthesis pathway, particularly in proposed diverse and expanded roles of key enzymes branching from anthocyanin pathway and in successful isolation of extension units for PA polymerization, but questions still need to be addressed to elucidate the mechanistical details of how PAs are synthesized, transported and regulated in various plant species. I envision that this will stimulate more studies and lead to new discoveries in this area.

## Author contributions

NL: Writing – original draft, Writing – review & editing.

## References

[B1] Alfenito M. R. Souer E. Goodman C. D. Buell R. Mol J. Koes R.. (1998). Functional complementation of anthocyanin sequestration in the vacuole by widely divergent glutathione S-transferases. Plant Cell. 10, 1135–1149. doi: 10.1105/tpc.10.7.1135 9668133 PMC144053

[B2] Amato A. Cavallini E. Zenoni S. Finezzo L. Begheldo M. Ruperti B.. (2017). A grapevine TTG2-like WRKY transcription factor is involved in regulating vacuolar transport and flavonoid biosynthesis. Front. Plant Sci. 7, 1979. doi: 10.3389/fpls.2016.01979 28105033 PMC5214514

[B3] An X. H. Tian Y. Chen K. Q. Liu X. J. Liu D. D. Xie X. B.. (2015). MdMYB9 and MdMYB11 are involved in the regulation of the JA-induced biosynthesis of anthocyanin and proanthocyanidin in apples. Plant Cell Physiol. 56, 650–662. doi: 10.1093/pcp/pcu205 25527830

[B4] Bhagwat S. Haytowitz D. B. (2015). USDA database for the proanthocyanidin content of selected foods, release 2 (Nutrient Data Laboratory, Beltsville Human Nutrition Research Center, ARS, USDA). doi: 10.15482/USDA.ADC/1324621

[B5] Bogs J. Downey M. O. Harvey J. S. Ashton A. R. Tanner G. J. Robinson S. P. (2005). Proanthocyanidin synthesis and expression of genes encoding leucoanthocyanidin reductase and anthocyanidin reductase in developing grape berries and grapevine leaves. Plant Physiol. 139, 652–663. doi: 10.1104/pp.105.064238 16169968 PMC1255985

[B6] Chen M. Xuan L. Wang Z. Zhou L. Li Z. Du X.. (2014). TRANSPARENT TESTA 8 inhibits seed fatty acid accumulation by targeting several seed development regulators in Arabidopsis. Plant Physiol. 165, 905–916. doi: 10.1104/pp.114.235507 24722549 PMC4044850

[B7] Cos P. De Bruyne T. Hermans N. Apers S. Berghe D. V. Vlietinck A. J. (2004). Proanthocyanidins in health care: current and new trends. Curr. Med. Chem. 11, 1345–1359. doi: 10.2174/0929867043365288 15134524

[B8] Cui X. Jun J. H. Rao X. Bahr C. Chapman E. Temple S.. (2022). Leaf layer-based transcriptome profiling for discovery of epidermal-selective promoters in *Medicago truncatula* . Planta 256, 31. doi: 10.1007/s00425-022-03920-4 35790623

[B9] Dixon R. A. Sarnala S. (2020). Proanthocyanidin biosynthesis—a matter of protection. Plant Physiol. 184, 579–591. doi: 10.1104/pp.20.00973 32817234 PMC7536678

[B10] Dixon R. A. Xie D. Y. Sharma S. B. (2005). Proanthocyanidins–a final frontier in flavonoid research? New Phytol. 165, 9–28. doi: 10.1111/j.1469-8137.2004.01217.x 15720617

[B11] Eichenberger M. Schwander T. Hüppi S. Kreuzer J. Mittl P. R. E. Peccati F.. (2023). The catalytic role of glutathione transferases in heterologous anthocyanin biosynthesis. Nat. Catal. 6, 927–938. doi: 10.1038/s41929-023-01018-y 37881531 PMC10593608

[B12] Furukawa T. Maekawa M. Oki T. Suda I. Iida S. Shimada H.. (2007). The *Rc* and *Rd* genes are involved in proanthocyanidin synthesis in rice pericarp. Plant J. 49, 91–102. doi: 10.1111/j.1365-313X.2006.02958.x 17163879

[B13] Gonzalez A. Brown M. Hatlestad G. Akhavan N. Smith T. Hembd A.. (2016). TTG2 controls the developmental regulation of seed coat tannins in Arabidopsis by regulating vacuolar transport steps in the proanthocyanidin pathway. Dev. Biol. 419, 54–63. doi: 10.1016/j.ydbio.2016.03.031 27046632

[B14] Hancock K. R. Collette V. Fraser K. Greig M. Xue H. Richardson K.. (2012). Expression of the R2R3-MYB transcription factor TaMYB14 from *Trifolium arvense* activates proanthocyanidin biosynthesis in the legumes *Trifolium repens* and *Medicago sativa* . Plant Physiol. 159, 1204–1220. doi: 10.1104/pp.112.195420 22566493 PMC3387705

[B15] Himi E. Taketa S. (2015). Barley Ant17, encoding flavanone 3-hydroxylase (F3H), is a promising target locus for attaining anthocyanin/proanthocyanidin-free plants without pleiotropic reduction of grain dormancy. Genome 58, 43–53. doi: 10.1139/gen-2014-0189 25932661

[B16] Jiao Y. Nigam D. Barry K. Daum C. Yoshinaga Y. Lipzen A.. (2023). A large sequenced mutant library - valuable reverse genetic resource that covers 98% of sorghum genes. Plant J. 117 (5), 1543–1557. doi: 10.1111/tpj.16582 38100514

[B17] Jonker A. Yu P. (2016). The role of proanthocyanidins complex in structure and nutrition interaction in alfalfa forage. Int. J. Mol. Sci. 17, 793. doi: 10.3390/ijms17050793 27223279 PMC4881609

[B18] Jun J. H. Liu C. Xiao X. Dixon R. A. (2015). The transcriptional repressor MYB2 regulates both spatial and temporal patterns of proanthocyanidin and anthocyanin pigmentation in *Medicago truncatula* . Plant Cell 27, 2860–2879. doi: 10.1105/tpc.15.00476 26410301 PMC4682322

[B19] Jun J. H. Lu N. Docampo-Palacios M. Wang X. Dixon R. A. (2021). Dual activity of anthocyanidin reductase supports the dominant plant proanthocyanidin extension unit pathway. Sci. Adv. 7, eabg4682. doi: 10.1126/sciadv.abg4682 33990337 PMC8121424

[B20] Jun J. H. Xiao X. Rao X. Dixon R. A. (2018). Proanthocyanidin subunit composition determined by functionally diverged dioxygenases. Nat. Plants 4, 1034–1043. doi: 10.1038/s41477-018-0292-9 30478357

[B21] Kitamura S. Matsuda F. Tohge T. Yonekura-Sakakibara K. Yamazaki M. Saito K.. (2010). Metabolic profiling and cytological analysis of proanthocyanidins in immature seeds of *Arabidopsis thaliana* flavonoid accumulation mutants. Plant J. 62, 549–559. doi: 10.1111/tpj.2010.62.issue-4 20180920

[B22] Kovinich N. Saleem A. Arnason J. T. Miki B. (2012). Identification of two anthocyanidin reductase genes and three red-brown soybean accessions with reduced anthocyanidin reductase 1 mRNA, activity, and seed coat proanthocyanidin amounts. J. Agric. Food Chem. 60, 574–584. doi: 10.1021/jf2033939 22107112

[B23] Liu C. Wang X. Shulaev V. Dixon R. A. (2016). A role for leucoanthocyanidin reductase in the extension of proanthocyanidins. Nat. Plants 2, 16182. doi: 10.1038/nplants.2016.182 27869786

[B24] Lu N. Jun J. H. Li Y. Dixon R. A. (2023). An unconventional proanthocyanidin pathway in maize. Nat. Commun. 14, 4349. doi: 10.1038/s41467-023-40014-5 37468488 PMC10356931

[B25] Lu N. Jun J. H. Liu C. G. Dixon R. A. (2022). The flexibility of proanthocyanidin biosynthesis in plants. Plant Physiol. 190, 202–205. doi: 10.1093/plphys/kiac274 35695780 PMC9434147

[B26] Lu N. Rao X. Li Y. Jun J. H. Dixon R. A. (2021). Dissecting the transcriptional regulation of proanthocyanidin and anthocyanin biosynthesis in soybean (*Glycine max*). Plant Biotech. J. 19, 1429–1442. doi: 10.1111/pbi.13562 PMC8313137 33539645

[B27] Lu N. Roldan M. Dixon R. A. (2017). Characterization of two TT2-type MYB transcription factors regulating proanthocyanidin biosynthesis in tetraploid cotton, *Gossypium hirsutum* . Planta 246, 323–335. doi: 10.1007/s00425-017-2682-z 28421329

[B28] Marrs K. A. Alfenito M. R. Lloyd A. M. Walbot V. (1995). A glutathione S-transferase involved in vacuolar transfer encoded by the maize gene Bronze-2. Nature 375, 397–400. doi: 10.1038/375397a0 7760932

[B29] McLaughlin M. Walbot V. (1987). Cloning of a mutable *bz2* allele of maize by transposon tagging and differential hybridization. Genetics 117, 771–776. doi: 10.1093/genetics/117.4.771 2828160 PMC1203248

[B30] Mei J. Niu Q. Xu K. Huang Y. Bai S. Zhu J.. (2023). GhmiR858 inhibits the accumulation of proanthocyanidins by targeting *GhTT2L* in cotton (*Gossypium hirsutum*). J. Agric. Food Chem. 71, 15341–15351. doi: 10.1021/acs.jafc.3c03884 37787767

[B31] Nesi N. Debeaujon I. Jond C. Pelletier G. Caboche M. Lepiniec L. (2000). The *TT8* gene encodes a basic helix-loop-helix domain protein required for expression of *DFR* and *BAN* genes in Arabidopsis siliques. Plant Cell 12, 1863–1878. doi: 10.1105/tpc.12.10.1863 11041882 PMC149125

[B32] Olsen O. Wang X. von Wettstein D. (1993). Sodium azide mutagenesis: preferential generation of A.T–>G.C transitions in the barley Ant18 gene. Proc. Natl. Acad. Sci. U.S.A. 90, 8043–8047. doi: 10.1073/pnas.90.17.8043 8367460 PMC47284

[B33] Roldan M. B. Cousins G. Muetzel S. Zeller W. E. Fraser K. Salminen J. P.. (2022). Condensed tannins in white clover (*Trifolium repens*) foliar tissues expressing the transcription factor TaMYB14-1 bind to forage protein and reduce ammonia and methane emissions in *vitro* . Front. Plant Sci. 12, 777354. doi: 10.3389/fpls.2021.777354 35069633 PMC8774771

[B34] Shoeva O. Y. Mukhanova M. A. Zakhrabekova S. Hansson M. (2023). Ant13 encodes regulatory factor WD40 controlling anthocyanin and proanthocyanidin synthesis in barley (*Hordeum vulgare* L.). J. Agric. Food Chem. 71, 6967–6977. doi: 10.1021/acs.jafc.2c09051 37104658

[B35] Tian Y. Liu X. Fan C. Li T. Qin H. Li X.. (2021). Enhancement of tobacco (*Nicotiana tabacum* L.) seed lipid content for biodiesel production by CRISPR-Cas9-mediated knockout of NtAn1. Front. Plant Sci. 11, 599474. doi: 10.3389/fpls.2020.599474 33552096 PMC7859101

[B36] Vale M. Rodrigues J. Badim H. Gerós H. Conde A. (2021). Exogenous application of non-mature miRNA-encoded miPEP164c inhibits proanthocyanidin synthesis and stimulates anthocyanin accumulation in grape berry cells. Front. Plant Sci. 12, 706679. doi: 10.3389/fpls.2021.706679 34675946 PMC8523857

[B37] Waghorn G. (2008). Beneficial and detrimental effects of dietary condensed tannins for sustainable sheep and goat production—Progress and challenges. Anim. Feed Sci. Technol. 147, 116–139. doi: 10.1016/j.anifeedsci.2007.09.013

[B38] Wang P. Liu Y. Zhang L. Wang W. Hou H. Zhao Y.. (2020). Functional demonstration of plant flavonoid carbocations proposed to be involved in the biosynthesis of proanthocyanidins. Plant J. 101, 18–36. doi: 10.1111/tpj.14515 31454118

[B39] Wang W. Q. Liu X. F. Zhu Y. J. Zhu J. Z. Liu C. Wang Z. Y.. (2023). Identification of miRNA858 long-loop precursors in seed plants. Plant Cell, koad315. doi: 10.1093/plcell/koad315 38114096 PMC11062470

[B40] Wu Y. Li X. Xiang W. Zhu C. Lin Z. Wu Y.. (2012). Presence of tannins in sorghum grains is conditioned by different natural alleles of *Tannin1* . Proc. Natl. Acad. Sci. U.S.A. 109, 10281–10286. doi: 10.1073/pnas.1201700109 22699509 PMC3387071

[B41] Xie D. Y. Sharma S. B. Paiva N. L. Ferreira D. Dixon R. A. (2003). Role of anthocyanidin reductase, encoded by BANYULS in plant flavonoid biosynthesis. Science 299, 396–399. doi: 10.1126/science.1078540 12532018

[B42] Xie D. Y. Dixon R. A. (2005). Proanthocyanidin biosynthesis–still more questions than answers? Phytochemistry 66, 2127–2144. doi: 10.1016/j.phytochem.2005.01.008 16153412

[B43] Xie Y. Ma Y. Bi P. Wei W. Liu J. Hu Y.. (2020). Transcription factor FvTCP9 promotes strawberry fruit ripening by regulating the biosynthesis of abscisic acid and anthocyanins. Plant Physiol. Biochem. 146, 374–383. doi: 10.1016/j.plaphy.2019.11.004 31794898

[B44] Yan Q. Wang Y. Li Q. Zhang Z. Ding H. Zhang Y.. (2018). Up-regulation of GhTT2-3A in cotton fibres during secondary wall thickening results in brown fibres with improved quality. Plant Biotechnol. J. 16, 1735–1747. doi: 10.1111/pbi.12910 29509985 PMC6131414

[B45] Yang B. Wei Y. Liang C. Guo J. Niu T. Zhang P.. (2022). VvANR silencing promotes expression of VvANS and accumulation of anthocyanin in grape berries. Protoplasma 259, 743–753. doi: 10.1007/s00709-021-01698-y 34448083

[B46] Yang S. Zhang M. Xu L. Luo Z. Zhang Q. (2020). MiR858b Inhibits proanthocyanidin accumulation by the repression of *DkMYB19* and *DkMYB20* in persimmon. Front. Plant Sci. 11, 576378. doi: 10.3389/fpls.2020.576378 33408726 PMC7779590

[B47] Yu K. Dixon R. A. Duan C. (2022). A role for ascorbate conjugates of (+)-catechin in proanthocyanidin polymerization. Nat. Commun. 13, 3425. doi: 10.1038/s41467-022-31153-2 35701431 PMC9197940

[B48] Zhai Y. Yu K. Cai S. Hu L. Amoo O. Xu L.. (2020). Targeted mutagenesis of *BnTT8* homologs controls yellow seed coat development for effective oil production in *Brassica napus* L. Plant Biotechnol. J. 18, 1153–1168. doi: 10.1111/pbi.13281 31637846 PMC7152602

[B49] Zhang B. Yang H. J. Qu D. Zhu Z. Z. Yang Y. Z. Zhao Z. Y. (2022). The MdBBX22-miR858-MdMYB9/11/12 module regulates proanthocyanidin biosynthesis in apple peel. Plant Biotechnol. J. 20, 1683–1700. doi: 10.1111/pbi.13839 35527510 PMC9398380

[B50] Zhu Y. Wang H. Peng Q. Tang Y. Xia G. Wu J.. (2015). Functional characterization of an anthocyanidin reductase gene from the fibers of upland cotton (*Gossypium hirsutum*). Planta 241, 1075–1089. doi: 10.1007/s00425-014-2238-4 25575669

[B51] Zhu Y. Yuzuak S. Sun X. Xie D. Y. (2023). Identification and biosynthesis of plant papanridins, a group of novel oligomeric flavonoids. Mol. Plant 16, 1773–1793. doi: 10.1016/j.molp.2023.09.015 37749887

